# Predicting Dementia Onset with the Updated Cut‐off Points of the National Center for Geriatrics and Gerontology‐Functional Assessment Tool

**DOI:** 10.1002/alz70860_100797

**Published:** 2025-12-23

**Authors:** Osamu Katayama, Ryo Yamaguchi, Daiki Yamagiwa, Shoma Akaida, Hiroyuki Shimada

**Affiliations:** ^1^ National Center for Geriatrics and Gerontology, Obu, Aichi, Japan

## Abstract

**Background:**

Early detection of mild cognitive impairment is important for preventing dementia. We have developed the National Center for Geriatrics and Gerontology‐Functional Assessment Tool, which can determine mild cognitive impairment by defining objective cognitive impairment based on age and education level. To clarify the relationship between the 1.5 standard deviations new cut‐off points for each cognitive test of the National Center for Geriatrics and Gerontology‐Functional Assessment Tool and incident of dementia.

**Method:**

The 2,441 participants without dementia at baseline were included in our longitudinal analysis for a monthly follow‐up of dementia onset. We monitored the incidence of dementia over a five‐year period using data from Public Health Insurance and the National Long‐Term Care Insurance records. We measured cognitive function using the National Center for Geriatrics and Gerontology‐Functional Assessment Tool and the Mini‐Mental State Examination.

**Result:**

Of the 2,441 study participants, 241 (9.9%) developed dementia during the 5‐year study period. The incidence rate of dementia was 21.7 per 1000 person‐years (95% CI: 19.1‐24.6). In Cox proportional hazard regression of each cognitive test, the hazard regression and 95% CI for those with a decline of 1.5 SD or more in word list memory, TMT‐A, forward digit span, backward digit span, TMT‐B, and SDST were 1.77 (95% CI: 1.28‐2.44), 1.37 (95% CI: 0.96‐1.95), 1.91 (95% CI: 1.29‐2.81), 2.12 (95% CI: 1.49‐3.02), 2.41 (95% CI: 1.78‐3.25), and 3.22 (95% CI: 2.39‐4.33), respectively. In Cox proportional hazard regression of cognitive state, the hazard regression and 95% CI for those with aMCIs, aMCIm, naMCIs, naMCIm, and GCI were 1.58 (95% CI: 0.86‐2.90), 2.17 (95% CI: 1.18‐4.00), 1.55 (95% CI: 1.04‐2.32), 2.92 (95% CI: 1.87‐4.58), and 2.29 (95% CI: 1.62‐3.25), respectively.

**Conclusion:**

The National Center for Geriatrics and Gerontology‐Functional Assessment Tool is an effective screening test for the early detection of dementia risk in community‐dwelling older adults.

